# Supplementary material for: Poor coherence in older people's speech is explained by impaired semantic and executive processes
Source: eLife. 2018 Sep 4;7:e38907. doi: 10.7554/eLife.38907 (PMC6150697; doi:10.7554/eLife.38907)
Supplement: Supplementary file 1. [file elife-38907-supp1.docx]

*Results of mixed effects models predicting characteristics of speech*

|  | Model 1 | | | Model 2 | | | Model 3 | | |
| --- | --- | --- | --- | --- | --- | --- | --- | --- | --- |
|  | *B* | *se* | *p* | *B* | *se* | *p* | *B* | *se* | *p* |
| *Factor 1: Vocabulary* |  |  |  |  |  |  |  |  |  |
| (Intercept) | -.002 | .195 | >.05 | -.002 | .195 | >.05 | -.002 | .195 | >.05 |
| Age | **.114** | **.042** | **.009** | **.117** | **.042** | **.009** | -.009 | .067 | .89 |
| Task | -.019 | .022 | .38 | -.019 | .022 | .38 | -.020 | .022 | .37 |
| Age*Task | -.008 | .022 | .70 | -.008 | .022 | .70 | -.009 | .022 | .69 |
| Response length | **-.108** | **.033** | **.001** | **-.107** | **.033** | **.002** | **-.114** | **.033** | **.001** |
| Trails ratio |  |  |  | -.014 | .041 | .73 | .001 | .039 | .98 |
| Semantic knowledge |  |  |  |  |  |  | **.167** | **.069** | **.017** |
| Semantic selection |  |  |  |  |  |  | -.006 | .039 | .89 |
| Weak association |  |  |  |  |  |  | -.017 | .049 | .73 |
| *Factor 2: Coherence* |  |  |  |  |  |  |  |  |  |
| (Intercept) | .000 | .151 | >.05 | .000 | .149 | >.05 | .001 | .148 | >.05 |
| Age | **-.295** | **.063** | **<.001** | **-.266** | **.061** | **<.001** | -.093 | .086 | .29 |
| Task | -.022 | .029 | .45 | -.022 | .029 | .45 | -.021 | .029 | .49 |
| Age*Task | -.054 | .029 | .078 | -.054 | .029 | .075 | -.054 | .029 | .076 |
| Response length | -.057 | .041 | .17 | -.051 | .040 | .21 | -.044 | .039 | .27 |
| Trails ratio |  |  |  | **-.142** | **.052** | **.009** | **-.156** | **.049** | **.002** |
| Semantic knowledge |  |  |  |  |  |  | **-.216** | **.085** | **.013** |
| Semantic selection |  |  |  |  |  |  | **.134** | **.049** | **.008** |
| Weak association |  |  |  |  |  |  | .028 | .061 | .64 |

| *Factor 3: Semantic specificity* | | | | | | | | | |
| --- | --- | --- | --- | --- | --- | --- | --- | --- | --- |
| (Intercept) | -.002 | .151 | >.05 | -.002 | .151 | >.05 | -.002 | .150 | >.05 |
| Age | .011 | .050 | .83 | .008 | .050 | .88 | .108 | .077 | .17 |
| Task | -.011 | .031 | .73 | -.011 | .031 | .73 | -.010 | .031 | .75 |
| Age*Task | -.018 | .034 | .60 | -.018 | .034 | .60 | -.018 | .034 | .60 |
| Response length | **-.125** | **.040** | **.002** | **-.127** | **.040** | **.002** | **-.115** | **.040** | **.004** |
| Trails ratio |  |  |  | .013 | .046 | .78 | .008 | .045 | .86 |
| Semantic knowledge |  |  |  |  |  |  | -.071 | .079 | .37 |
| Semantic selection |  |  |  |  |  |  | .070 | .045 | .12 |
| Weak association |  |  |  |  |  |  | -.065 | .056 | .25 |
| *Factor 4: Lexical diversity* | | | | | | | | | |
| (Intercept) | -.001 | .131 | >.05 | -.001 | .131 | >.05 | -.002 | .129 | >.05 |
| Age | .143 | .080 | .078 | .138 | .081 | .094 | -.100 | .119 | .41 |
| Task | -.028 | .025 | .26 | -.028 | .025 | .26 | -.028 | .025 | .26 |
| Age*Task | .012 | .029 | .68 | -.012 | .029 | .68 | -.012 | .029 | .68 |
| Response length | **-.311** | **.044** | **<.001** | **-.312** | **.044** | **<.001** | **-.318** | **.043** | **<.001** |
| Trails ratio |  |  |  | .025 | .071 | .73 | .047 | .068 | .49 |
| Semantic knowledge |  |  |  |  |  |  | **.276** | **.120** | **.024** |
| Semantic selection |  |  |  |  |  |  | -.059 | .068 | .39 |
| Weak association |  |  |  |  |  |  | .019 | .086 | .68 |
